# The British Nursing Profession: The Two Voices

**Published:** 1917-09-15

**Authors:** 


### The British Nursing Profession: The Two Voices.

*To the Editor of* THE HOSPITAL.

SIR,—To one carefully reading the various letters appearing under the title "Matrons in Council," it becomes apparent that there are two voices speaking—one is concerned with the question of education alone (taking the word in its restricted sense of training-school examinations), the other voice would bring forward the claim of the worker in her post-hospital life, as the most pressing need of the moment. Both these voices have stated their case very briefly; of course in short articles such as these, which must necessarily be restricted as to space, it is impossible for either to do more than indicate the weak points in the general structure which call for attention. However, enough has been outlined to indicate at least that there do exist undoubtedly certain burning questions which can be advanced by discussion in the press, in preparation for their practical settlement at meetings of those in a position to deal with these important matters.

No doubt many readers of THE HOSPITAL have read and remember a wonderful story by Rudyard Kipling, called "The Ship which Found Herself," which shows the absolute necessity in promoting combined movement for the sound of many voices, at first separate, astonished, even angry, but finally as a wider and greater understanding dawns (the natural result of work together, where each has to give and take), the tones of the voices change and merge one into the other in a strange and natural harmony, making ONE voice, the voice of the whole ship. In the present discussion in THE HOSPITAL we are hearing the many voices, in time we shall hear the one composite voice if we have patience.

There is no doubt that matrons have worked and do work hard to keep up the standard of education as decided upon, and there is no doubt also that they have many difficulties to contend with which are not easily understood by others. Moreover, there are certain posts where the title of matron is used by those who ought not so to use it, and this brings confusion to outsiders; equally there are great and unnecessary hardships in the lives of nurses after they leave their training-schools and pass to their work in the greater world outside. To an Onlooker it appears that it is the hardships and want of protection in the lives of these workers which is the great point requiring consideration at this moment; it would seem that what the second Voice asks of the first Voice is help in this direction.

These are some of the points which might be brought forward :

(1) How many hours do nurses work in the twenty-four? Is there a regular number for a certain wage, and is anything over reckoned as overtime?

(2) What commission are nurses required to pay the various Associations who employ them?

(3) Do these commission fees do more than cover the legitimate clerical working of such Associations, and, if so, how is the surplus in each case used? Could not greater economy in working be obtained by combining agencies?

(4) Is it a fact that numerous nursing homes are at least partially staffed by untrained women who receive a small salary, but whose services are paid for by patients at the rate of a trained nurse?

One correspondent suggests that home training is a very necessary factor in the equipment of a nurse; one concedes this most heartily, but that very fact makes it the more urgent that post-training legislation should be taken in hand; women brought up in well-regulated homes cannot be expected to put up with the often rough living, the lack of privacy, particularly in sleeping arrangements, which is allotted to them in a large number of so-called nursing homes and elsewhere. It must mean that only a rougher type of woman will undertake that work, and this, in the long run, will militate against, and, let us hope, secure the closing of the inefficient nursing homes.

There is certainly dissatisfaction, and since the coming of Canada, Australia, and America these questions have become more acute. The second Voice would, therefore, appear to be right in urging that this point of view needs the earliest and prompt remedial attention.

This Onlooker who writes has been made aware of many things in carefully following these articles; it is open to others likewise to investigate what is here written, and much that appears every week in THE HOSPITAL.—Yours faithfully,

